# Hybrid coronary revascularization vs. PCI in high-risk multivessel coronary artery disease: a two-center, two-year utcome comparison

**DOI:** 10.3389/fcvm.2025.1661189

**Published:** 2025-12-01

**Authors:** Ting Luo, Dong Yi, Lingzhi Qiu, Zhengle Yang, Xiaodie Xu, Dan Song, Zhengdong Hua, Xufa Chen, Bingyin Wang, Hua Yan

**Affiliations:** 1 Department of Cardiology, Wuhan Asia Heart Hospital, Wuhan, China; 2 Department of Cardiac Surgery, Wuhan Asia Heart Hospital, Wuhan, China; 3 Department of Cardiology, Wuhan Asia General Hospital, Wuhan, China

**Keywords:** hybrid coronary revascularization, percutaneous coronary intervention, multivessel coronary artery disease, MACCE, outcomes

## Abstract

**Background:**

Treatment strategies for multivessel coronary artery disease (MV-CAD) include percutaneous coronary intervention (PCI), coronary artery bypass grafting (CABG), and the increasingly adopted hybrid coronary revascularization (HCR). HCR combines minimally invasive left internal mammary artery (LIMA)–to–left anterior descending (LAD) grafting with PCI of non-LAD lesions. However, comparative evidence in high-risk MV-CAD remains limited.

**Methods:**

We retrospectively analyzed 330 high-risk MV-CAD patients from two centers (HCR *n* = 109; PCI *n* = 221) over 2 years. The primary endpoint was major adverse cardiac and cerebrovascular events (MACCE: all-cause death, stroke, myocardial infarction, repeat revascularization, and angina). Kaplan–Meier survival analysis and conventional statistical tests were applied.

**Results:**

Baseline demographics and SYNTAX scores were similar between groups. HCR involved fewer and shorter stents than PCI. Hospital stay, ICU duration, and total costs were higher with HCR. At 2 years, angina (5.5% vs. 17.2%; *P* = 0.003) and MACCE (12.8% vs. 23.5%; *P* = 0.02) were lower with HCR; overall survival by Kaplan–Meier favored HCR (log-rank *P* = 0.0006).

**Conclusions:**

Despite longer hospitalization and higher costs, HCR was associated with superior long-term symptom relief and lower MACCE compared with PCI in high-risk MV-CAD. These findings support HCR as a viable strategy in carefully selected patients and warrant validation in prospective multicenter studies.

## Introduction

Cardiovascular diseases remain the leading cause of mortality worldwide. In China, the number of coronary artery disease (CAD) patients exceeds 11 million (1). With population aging and a rising prevalence of metabolic comorbidities, the incidence of multivessel CAD (MV-CAD) is increasing. Treatment selection becomes particularly complex in patients with high-risk features, such as advanced age, diabetes, and impaired left ventricular function.

**Table 1:** Baseline clinical characteristics.

Baseline clinical characteristics	HCR group (*n* = 109)	PCI group (*n* = 221)	Statistic	*P* Value
Age, years	63.12 ± 8.31	62.44 ± 9.29	−0.64	0.52
Male sex	72 (66.1%)	148 (67.0%)	0.03	0.87
Body mass index, kg/m²	24.20 ± 3.44	24.10 ± 3.29	−0.24	0.80
Clinical classification—Stable angina	72 (66.1%)	135 (61.1%)	0.06	0.81
Clinical classification—NSTEMI	37 (33.9%)	86 (38.9%)	0.32	0.49
Smoking	47 (43.1%)	107 (48.4%)	0.82	0.36
Hypertension	76 (69.7%)	154 (69.7%)	0.00	0.99
Diabetes mellitus	38 (34.9%)	80 (36.2%)	0.06	0.81
Hyperlipidemia	44 (40.4%)	83 (37.6%)	0.24	0.62
Previous cerebrovascular accident	38 (34.9%)	58 (26.2%)	2.63	0.11
Previous MI	12 (11.0%)	36 (16.3%)	1.64	0.20
Previous PCI	8 (7.3%)	24 (10.9%)	1.03	0.31
Previous CABG	0 (0)	0 (0)	–	–
During hospitalization—cTnI, ng/mL	0.02 (0.01, 0.19)	0.03 (0.01, 0.90)	−0.29	0.77
Creatinine, µmol/L	79.82 ± 24.34	81.60 ± 30.08	0.53	0.59
GFR, mL/min	84.25 ± 19.77	84.68 ± 20.57	0.18	0.86
Maximum hs-CRP, mg/L	3.19 (0.94, 37.50)	2.97 (0.92, 10.92)	−1.47	0.14
Maximum NT-proBNP, pg/mL	552.20 (187.30, 993)	283.75 (115.25, 1,141.68)	−0.82	0.42
Echo—LVD, cm	4.83 ± 0.52	4.98 ± 0.59	2.19	0.03
Echo—LVEF, %	53.42 ± 6.97	51.19 ± 8.27	−2.41	0.02

Contemporary revascularization strategies include PCI, CABG, and hybrid coronary revascularization (HCR) (2, 3). According to the 2018 ESC/EACTS Guidelines on myocardial revascularization (4), CABG is preferred in patients with complex anatomy or diabetes, whereas PCI is reasonable for less complex anatomy or when surgical risk is high. HCR integrates minimally invasive LIMA-to-LAD bypass with PCI for non-LAD lesions, and is often considered for left main disease, three-vessel disease, chronic total occlusions, heavy calcification, and bifurcation lesions (5, 6).

Despite its theoretical appeal, HCR adoption is limited by procedural complexity, the need for multidisciplinary coordination, and institutional experience. Most prior reports are single-center with small samples or short follow-up (7–11). Here, we compare 2-year outcomes of HCR vs. PCI in high-risk MV-CAD using real-world data from two centers.

## Materials and methods

### Study design and population

This retrospective, two-center observational study included 330 high-risk MV-CAD patients treated between November 2008 and February 2022. Patients were assigned to HCR (*n* = 109) or PCI (*n* = 221) according to the strategy received.

### High-risk definition

High-risk MV-CAD was defined by anatomical and/or clinical criteria: left main disease, three-vessel disease, chronic total occlusion (CTO), severe calcification, complex bifurcation or tortuous lesions, diabetes, or reduced left ventricular function. In addition, SYNTAX II–predicted 4-year mortality was calculated for each patient (HCR 10.6% ± 3.4; PCI 10.2% ± 3.1). Treatment strategy was determined by a multidisciplinary Heart Team after comprehensive assessment of safety and risk.

### HCR strategy

Staged HCR combined minimally invasive direct coronary artery bypass (MIDCAB) for LAD revascularization with PCI to non-LAD vessels (12–14). Among HCR patients, 37 underwent PCI first and 72 underwent MIDCAB first; the interval between procedures was ≤30 days (mean 10 ± 5 days). When MIDCAB was performed first, dual antiplatelet therapy (DAPT) was not discontinued; before MIDCAB after PCI, clopidogrel was held for 3 days and aspirin was continued; DAPT was resumed post-procedure.

### Inclusion criteria

(1) Age ≥18 years; (2) Angiographic diagnosis of MV-CAD (LAD plus ≥1 major non-LAD epicardial vessel); (3) At least one high-risk feature as defined above; (4) Heart-Team–based decision-making.

### Exclusion criteria

(1) STEMI patients requiring emergent primary PCI; (2) Pregnancy or breastfeeding; (3) Active malignancy or life expectancy <2 years; (4) Severe hepatic or renal dysfunction; (5) Prior CABG; (6) Contraindications to antiplatelet therapy; (7) Incomplete follow-up or missing data.

### Endpoints

The primary endpoint was 2-year MACCE (all-cause mortality, non-fatal MI per the Fourth Universal Definition (15), ischemic stroke confirmed by neuroimaging, repeat revascularization, and clinically diagnosed angina based on CCS class and nitroglycerin response).

### Statistical analysis

Continuous variables are presented as mean ± SD or median (IQR) and were compared using t tests or Wilcoxon rank-sum tests, as appropriate. Categorical variables are presented as *n* (%) and were compared using chi-square or Fisher's exact tests. Kaplan–Meier curves were compared using the log-rank test. Two-sided *P* < 0.05 was considered statistically significant.

### Ethics

The study complied with the Declaration of Helsinki and was approved by the Ethics Committee of Wuhan Asia Heart Hospital (Approval No.: 2025-B028). Informed consent was obtained from all patients.

## Results

### Study cohort

We included 330 patients (HCR *n* = 109; PCI *n* = 221). In the HCR group, 72 had stable angina and 37 had acute coronary syndrome (ACS); in the PCI group, 135 had stable angina and 86 had ACS. Baseline demographics (age 63.1 ± 8.3 vs. 62.4 ± 9.3 years; male 66.1% vs. 67.0%) and SYNTAX scores were similar between groups (Tables 1, 2).

### Procedural profile

HCR used fewer stents (1.93 ± 0.86 vs. 3.09 ± 1.22) and shorter total stent length (50.98 ± 26.48 mm vs. 77.83 ± 34.05 mm; both *P* < 0.001). PCI procedure time was longer in the PCI group (69.26 ± 32.19 vs. 46.63 ± 26.79 min; *P* < 0.001) (Table 2).

**Table 2:** Lesion and procedural characteristics.

Lesion and procedural characteristics	HCR group (*n* = 109)	PCI group (*n* = 221)	Statistic	*P* Value
Left Main (LM)	18 (16.5%)	31 (14.0%)	0.36	0.55
Left Anterior Descending (LAD)	109 (100%)	221 (100%)	–	–
Left Circumflex (LCX)	94 (86.2%)	190 (86.4%)	0.001	0.98
Right Coronary Artery (RCA)	98 (89.9%)	191 (86.4%)	0.81	0.37
LM + three-vessel disease	18 (16.5%)	19 (8.6%)	4.59	0.03
Three-vessel disease	65 (59.6%)	151 (68.3%)	2.44	0.12
Two-vessel disease	26 (23.9%)	51 (23.2%)	0.02	0.89
Chronic total occlusion	39 (35.8%)	89 (40.3%)	0.62	0.43
Severe calcification	34 (31.2%)	74 (33.5%)	0.17	0.68
Complex tortuous lesion	8 (7.3%)	30 (13.6%)	2.79	0.10
Bifurcation lesion	35 (32.1%)	66 (29.9%)	0.17	0.68
SYNTAX score	32.52 ± 5.59	32.38 ± 4.96	−0.23	0.81
SYNTAX II 4-year mortality, %	10.6 ± 3.4	10.2 ± 3.1	0.36	0.72
PCI time, min	46.63 ± 26.79	69.26 ± 32.19	6.30	<0.001
Implanted stents, n	1.93 ± 0.86	3.09 ± 1.22	8.92	<0.001
Total stent length, mm	50.98 ± 26.48	77.83 ± 34.05	7.20	<0.001

### Periprocedural and postoperative markers

Postoperative cTnI was lower in HCR; left ventricular diameter (LVD) was smaller and LVEF modestly higher in HCR. Renal function and inflammatory markers did not differ materially between groups (Table 3).

**Table 3:** Postoperative clinical characteristics.

Postoperative clinical characteristics	HCR Group (*n* = 109)	PCI Group (*n* = 221)	Statistic	*P* Value
Cardiac Troponin I (cTnI), ng/mL	0.06 (0.02, 0.32)	0.22 (0.05, 1)	−3.53	<0.001
Creatinine, µmol/L	84.41 ± 27.89	87.11 ± 34.53	0.68	0.50
GFR, mL/min	80.41 ± 21.05	79.31 ± 21.85	−0.42	0.68
LVD, cm	4.62 ± 0.44	4.93 ± 0.53	5.11	<0.001
LVEF, %	53.75 ± 6.06	52.65 ± 7.14	−1.34	0.18

### Economics

Length of stay (22.64 ± 6.64 vs. 7.93 ± 3.15 days), ICU stay, and total hospitalization costs (13.72 ± 3.85 vs. 6.27 ± 3.07 × 10,000 CNY) were higher with HCR (all *P* < 0.001) (Table 4).

**Table 4:** Economic indicators.

Economic indicators	HCR group (*n* = 109)	PCI group (*n* = 221)	Statistic	*P* Value
Length of hospital stay, days	22.64 ± 6.64	7.93 ± 3.15	−27.31	<0.001
Total hospitalization costs, ×10,000 CNY	13.72 ± 3.85	6.27 ± 3.07	−18.45	<0.001
Postoperative ICU stay, hours	48 (40.50, 72)	24 (14.25, 72)	−5.49	<0.001

### Clinical outcomes

**Figure 1:**
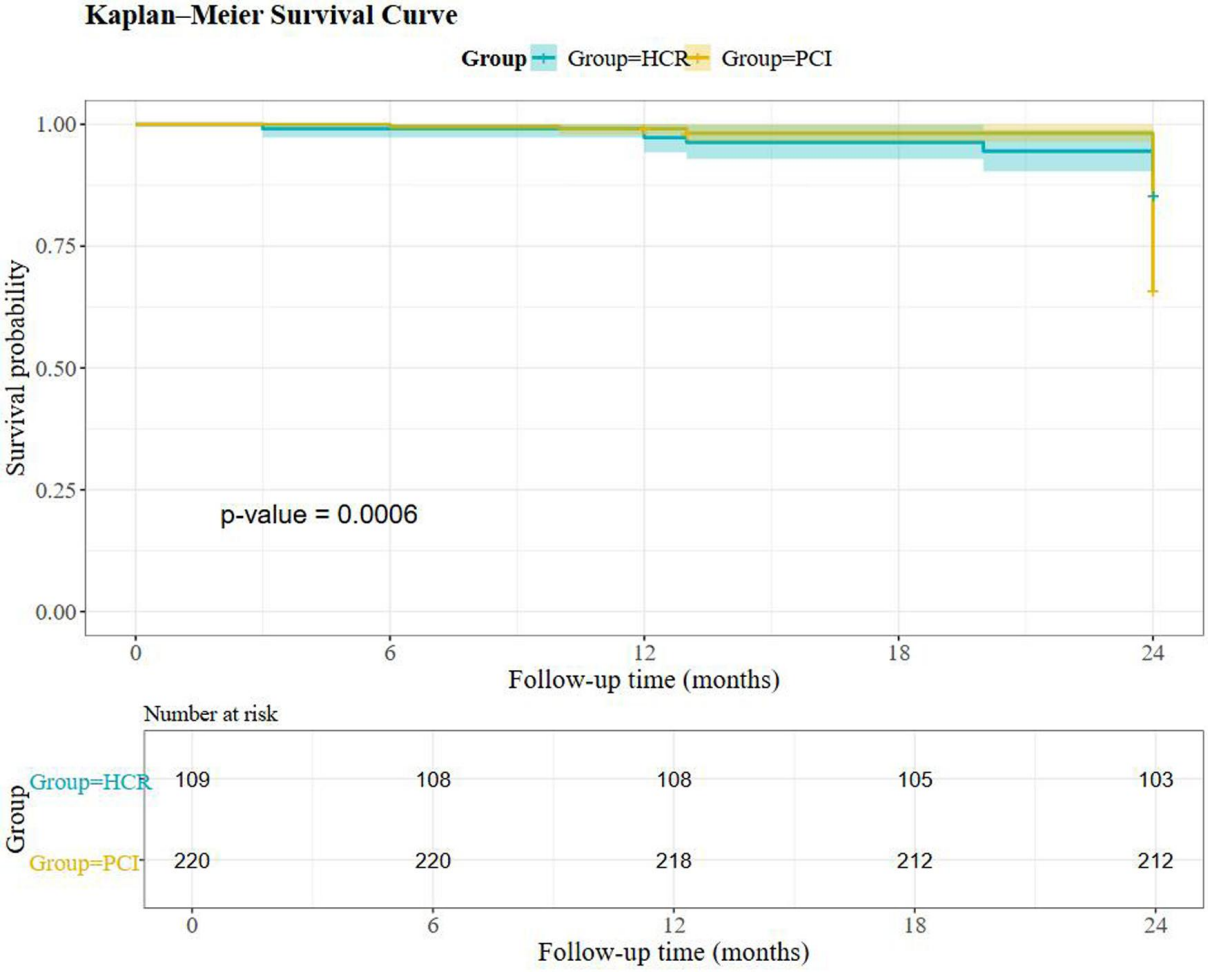
Kaplan–Meier survival curves comparing HCR and PCI (with number-at-risk table).

MACCE did not differ at 3 months or 1 year. At 2 years, HCR had lower angina (5.5% vs. 17.2%; *P* = 0.003) and MACCE (12.8% vs. 23.5%; *P* = 0.02) (Table 5). Overall survival favored HCR by Kaplan–Meier analysis (log-rank *P* = 0.0006) (Figure 1).

**Table 5:** Clinical outcomes.

Outcome	HCR group (*n* = 109)	PCI group (*n* = 221)	Statistic	*P* Value
MACCE at 3 months	5 (4.6%)	15 (6.8%)	0.62	0.43
All-cause mortality	2 (1.8%)	2 (0.9%)	0.53	0.47
Stroke	0 (0)	0 (0)	–	–
Myocardial infarction	1 (0.9%)	2 (0.9%)	0.00	>0.99
Repeat revascularization	0 (0)	3 (1.4%)	1.49	0.22
Angina symptoms	2 (1.8%)	14 (6.3%)	3.20	0.07
MACCE at 1 year	9 (8.3%)	26 (11.8%)	0.95	0.33
All-cause mortality	3 (2.8%)	5 (2.3%)	0.07	0.79
Stroke	0 (0)	0 (0)	–	–
Myocardial infarction	1 (0.9%)	5 (2.3%)	0.74	0.39
Repeat revascularization	1 (0.9%)	5 (2.3%)	0.74	0.39
Angina symptoms	5 (4.6%)	20 (9.0%)	2.08	0.15
MACCE at 2 years	14 (12.8%)	52 (23.5%)	5.21	0.02
All-cause mortality	7 (6.4%)	12 (5.4%)	0.13	0.72
Stroke	1 (0.9%)	2 (0.9%)	0.00	>0.99
Myocardial infarction	1 (0.9%)	7 (3.2%)	1.56	0.21
Repeat revascularization	1 (0.9%)	9 (4.1%)	2.47	0.12
Angina symptoms	6 (5.5%)	38 (17.2%)	8.63	0.003

## Discussion

In this real-world, two-center cohort of high-risk MV-CAD, HCR yielded lower 2-year angina and MACCE than PCI, despite longer hospitalization and higher costs. The combination of durable LIMA–LAD patency with reduced stent burden in non-LAD vessels likely contributed to these findings.

Patients treated with PCI alone required more and longer stents, potentially increasing the risks of restenosis and stent-related events. Our observations align with prior randomized and observational studies reporting symptom reduction and fewer repeat interventions with HCR in complex disease subsets.

Safety of HCR in high-risk patients is a key consideration. In our cohort, serious perioperative complications were infrequent; for transparency, we added a summary table of complications (e.g., IABP use, major bleeding, prolonged ventilation), supporting the feasibility of staged HCR.

Importantly, the MACCE difference was driven mainly by angina reduction, highlighting HCR's effect on symptom control and quality of life. While encouraging, this pattern warrants cautious interpretation of prognostic benefit and underscores the need for adequately powered, prospective multicenter trials with longer follow-up and advanced adjustment methods (e.g., propensity matching or weighting).

Future directions include refined patient selection using comprehensive risk models (e.g., SYNTAX II/III), incorporation of physiology-guided PCI and intravascular imaging, and evaluation of cost-effectiveness (16–18). Advances in minimally invasive and robotic techniques may further enhance the precision and scalability of HCR.

## Conclusion

HCR is a feasible and effective option for selected high-risk MV-CAD patients, providing superior 2-year symptom relief and lower MACCE compared with PCI, albeit with greater resource use. These real-world data support broader evaluation of HCR in prospective, multicenter studies.

## Data Availability

The original contributions presented in the study are included in the article/Supplementary Material, further inquiries can be directed to the corresponding author.
